# Targeting triglycerides for cardiovascular risk reduction

**DOI:** 10.1007/s11739-026-04383-1

**Published:** 2026-05-18

**Authors:** Carlo Maiorca, Daniele Tramontano, Alessia Di Costanzo, Laura D’Erasmo

**Affiliations:** 1 Department of Translational and Precision Medicine, Sapienza University of Rome, Rome, Italy; 2 UOC Pronto Soccorso E Medicina d’Urgenza, San Filippo Neri Hospital, ASL Roma 1, Rome, Italy

**Keywords:** Triglyceride-rich lipoproteins (TRLs), Residual cardiovascular risk, Hypertriglyceridemia (HTG), TG-lowering therapies

## Abstract

Residual cardiovascular risk persists in many patients despite optimal control of established factors such as low-density lipoprotein cholesterol (LDL-C), blood pressure and glycaemia. Mounting evidence indicates that hypertriglyceridemia (HTG) and triglyceride-rich lipoproteins (TRLs), including their remnant particles, contribute to atherogenesis and therefore constitute actionable targets for risk reduction. This narrative review summarizes current knowledge of TRL metabolism and its role in atherosclerotic cardiovascular disease (ASCVD), drawing on mechanistic studies, observational cohorts and Mendelian-randomization analyses. TRLs display pro-inflammatory and pro-thrombotic properties and are increasingly implicated in plaque initiation and progression. Traditional and emerging therapeutic strategies designed to lower TRL burden are critically examined with particular emphasis on their ability to address residual cardiovascular risk. Conventional interventions, fibrates and mixed omega-3 fatty-acid formulations, have yielded modest and inconsistent cardiovascular benefits. By contrast, icosapent ethyl, a highly purified ethyl ester of eicosapentaenoic acid, is the only TRL-targeted therapy that has demonstrated a significant reduction in major cardiovascular events in a large, randomized trial, irrespective of baseline triglyceride levels. Novel agents such as evinacumab (an ANGPTL3 monoclonal antibody) and antisense oligonucleotides against apolipoprotein C-III (volanesorsen, olezarsen, plozasiran) have produced marked decreases in triglycerides, apoB-containing lipoproteins and remnant cholesterol in recent clinical studies, even though cardiovascular outcome data are not yet available. Therefore, therapies targeting TRL metabolism represent a promising approach to reduce residual cardiovascular risk, particularly in high-risk or statin-treated patients.

## Introduction

Cardiovascular disease (CVD) remains the leading cause of morbidity and mortality worldwide, driven by a complex interplay of modifiable and non-modifiable risk factors. Among the modifiable contributors, elevated levels of low-density lipoprotein cholesterol (LDL-C) play a central role in the pathogenesis of atherosclerosis and subsequent clinical events such as myocardial infarction and stroke [1]. However, despite intensive lipid-lowering therapy and achievement of guideline-recommended LDL-C targets, a substantial proportion of patients remain at high risk for cardiovascular events, a phenomenon known as residual cardiovascular risk [2]. This risk is increasingly recognized as multifactorial, involving elements such as elevated lipoprotein(a), chronic low-grade inflammation (e.g., high-sensitivity C-reactive protein), and persistent elevations in triglyceride-rich lipoproteins (TRLs) [2]. In this context, hypertriglyceridemia (HTG) has gained attention as a potential contributor to this risk, even though the underlying mechanisms are still being explored [3, 4].

HTG is a metabolic disorder characterized by elevated circulating triglyceride (TG) concentrations, typically defined as fasting plasma TG levels > 150 mg/dL (1.7 mmol/L). HTG may result from impaired clearance of TRLs, most often due to pathogenic variants in the lipolytic pathway, ranging from monogenic defects in *LPL* gene or its cofactors (*APOC2, APOA5, LMF1*, and *GPIHBP1*) to polygenic susceptibility, and may be exacerbated by secondary factors such as diabetes, obesity, alcohol use, and certain medications [5, 6].

HTG is a well-known risk factor for acute pancreatitis (AP), depending on TG levels, where the risk progressively increases with TG > 500 mg/dL and is markedly elevated when levels exceed 1000 mg/dL [7]. Moreover, TRLs and their remnants are increasingly recognized as causal drivers of atherosclerosis. Genetic, epidemiologic, and mechanistic studies have established their contribution to atherogenic risk, supporting TG lowering as a promising strategy to address residual cardiovascular risk [8].

The recent ESC/EAS 2025 Focused Update Guideline [9] emphasizes that elevated TG and TRLs contribute independently to cardiovascular risk, even in patients achieving LDL-C targets. These guidelines highlight the importance of assessing and managing hypertriglyceridemia within a comprehensive, integrated approach to risk reduction, which includes the identification of secondary causes, optimization of overall cardiometabolic control, and attention to TRLs as potential therapeutic targets.

In this review, we summarize the current evidence linking TRL metabolism to atherosclerotic cardiovascular disease and discuss the emerging approaches aimed at reducing triglyceride-related residual cardiovascular risk.

## Triglyceride-rich lipoproteins metabolism

TRLs, primarily chylomicrons (CM) and very low-density lipoproteins (VLDL), are secreted by the intestine and liver, respectively, and are the main vehicles for TG transport in the circulation. In the postprandial state, CM are assembled in enterocytes through the packaging of dietary TG, cholesterol, and apolipoprotein B48 [10]. In parallel, hepatocytes continuously secrete VLDL particles containing apolipoprotein B100, incorporating TG derived from de novo lipogenesis, circulating non-esterified fatty acids, hepatic lipid stores, and remnant uptake [10].

Once in the bloodstream, CM and VLDL undergo lipolysis mediated by lipoprotein lipase (LPL), which hydrolyzes their TG core and facilitates the release of free fatty acids for uptake by peripheral tissues, particularly skeletal and cardiac muscle for oxidation, and adipose tissue for storage. As a result of this process, CM are converted into chylomicron remnants, whereas VLDL particles are gradually transformed into intermediate-density lipoproteins (IDL) and, in part, into low-density lipoproteins (LDL) through further delipidation catalyzed by hepatic lipase [11]. The remnant particles generated by this sequence—chylomicron remnants, IDL, and a fraction of LDL—are subsequently cleared by the liver through specific receptor-mediated pathways, such as the low-density lipoprotein receptor (LDL-R), LDL receptor–related protein 1 (LRP1) and scavenger receptor class B type 1 (SR-B1), ensuring efficient hepatic uptake of cholesterol-enriched lipoproteins [11].

The balance between TRL production, peripheral lipolysis, and hepatic clearance defines the overall flux of circulating TG and determines the extent of remnant particle accumulation. Under physiological conditions, this system ensures coordinated lipid delivery to energy-demanding tissues and timely clearance of remnant particles. However, in metabolic disorders such as insulin resistance, type 2 diabetes, chronic kidney disease, and nephrotic syndrome, an imbalance between production and clearance of TRLs favors their accumulation, promoting a lipid profile characterized by elevated TG, increased remnant particles, and changes in lipoprotein composition often associated with atherogenic risk [12, 13].

## The role of TRLs in residual cardiovascular risk

Persistent elevations in TRLs have emerged as both markers and mediators of atherogenic processes, with growing evidence supporting their relevance from both biological and clinical perspectives. TRLs not only transport lipids but actively participate in a complex interplay of lipid retention, cellular activation, and vascular inflammation, all fundamental drivers of atherosclerotic disease progression [14, 15]. Atherogenicity of TRLs largely depends on the structural and functional properties of their remnant particles, which are smaller and enriched in cholesterol relative to their parental TRLs. Unlike large chylomicrons, whose size often exceeds 1000 nm and restricts transendothelial passage, triglyceride-rich lipoprotein remnants, including chylomicron remnants, VLDL remnants, and IDL, range in diameter from approximately 30 to 80 nm, enabling passage across the endothelial barrier [16]. Upon entering the arterial intima, these remnants are selectively retained through binding to extracellular matrix proteoglycans, prolonging their residence time in the subendothelial space. This retention facilitates uptake by resident macrophages via scavenger receptors, without requiring the oxidative modification typical of LDL particles. The elevated cholesterol content per remnant particle, compared to LDL, amplifies their contribution to lipid accumulation and foam cell formation, key early events in plaque development [17].

Endothelial-bound lipoprotein lipase (LPL) catalyzes the hydrolysis of triglycerides within TRLs, releasing free fatty acids and monoacylglycerols. These lipolysis products act as bioactive mediators that induce endothelial dysfunction and local inflammation, stimulating the production of proinflammatory cytokines such as tumor necrosis factor-alpha (TNF-α), interleukin-1 beta (IL-1β), and interleukin-6 (IL-6). The resulting cytokine milieu promotes endothelial activation and upregulation of adhesion molecules, including vascular cell adhesion molecule-1 (VCAM-1) and intercellular adhesion molecule-1 (ICAM-1), thereby facilitating monocyte recruitment into the arterial wall [18]. This enhances monocyte recruitment and transmigration into the arterial intima, establishing a localized inflammatory milieu that perpetuates atherosclerotic plaque progression and instability. Moreover, the sustained production of these cytokines not only perpetuates vascular inflammation but also influences systemic immune responses, linking metabolic dyslipidemia to chronic inflammatory states that exacerbate atherogenesis [19–21]. Systemically, TRLs and their remnants have been linked to markers of low-grade inflammation, including hs-CRP, circulating cytokines such as IL-6 and TNF-α, and immune cell activation [22–28]. However, evidence remains sparse, and the precise relationship between TRLs and systemic inflammatory biomarkers requires further clarification.

In addition, TRL remnants contribute to a prothrombotic phenotype within the vascular wall by promoting platelet activation and aggregation, and by modulating both endothelial and systemic hemostatic responses [29–32]. Specifically, they promote the expression of tissue factor on activated endothelium and monocyte-derived macrophages, initiating coagulation cascades at sites of vascular injury [33]. Moreover, remnants have been shown to increase the expression of plasminogen activator inhibitor-1 (PAI-1), the primary inhibitor of tissue plasminogen activator (tPA), thereby impairing fibrinolytic activity. Elevated PAI-1 levels lead to reduced clot resolution capacity and have been associated with enhanced thrombus persistence, particularly at sites of plaque disruption [29, 30]. These effects reinforce the link between remnant lipoproteins and the thrombo-inflammatory axis of atherosclerotic disease progression.

**Fig. 1:**
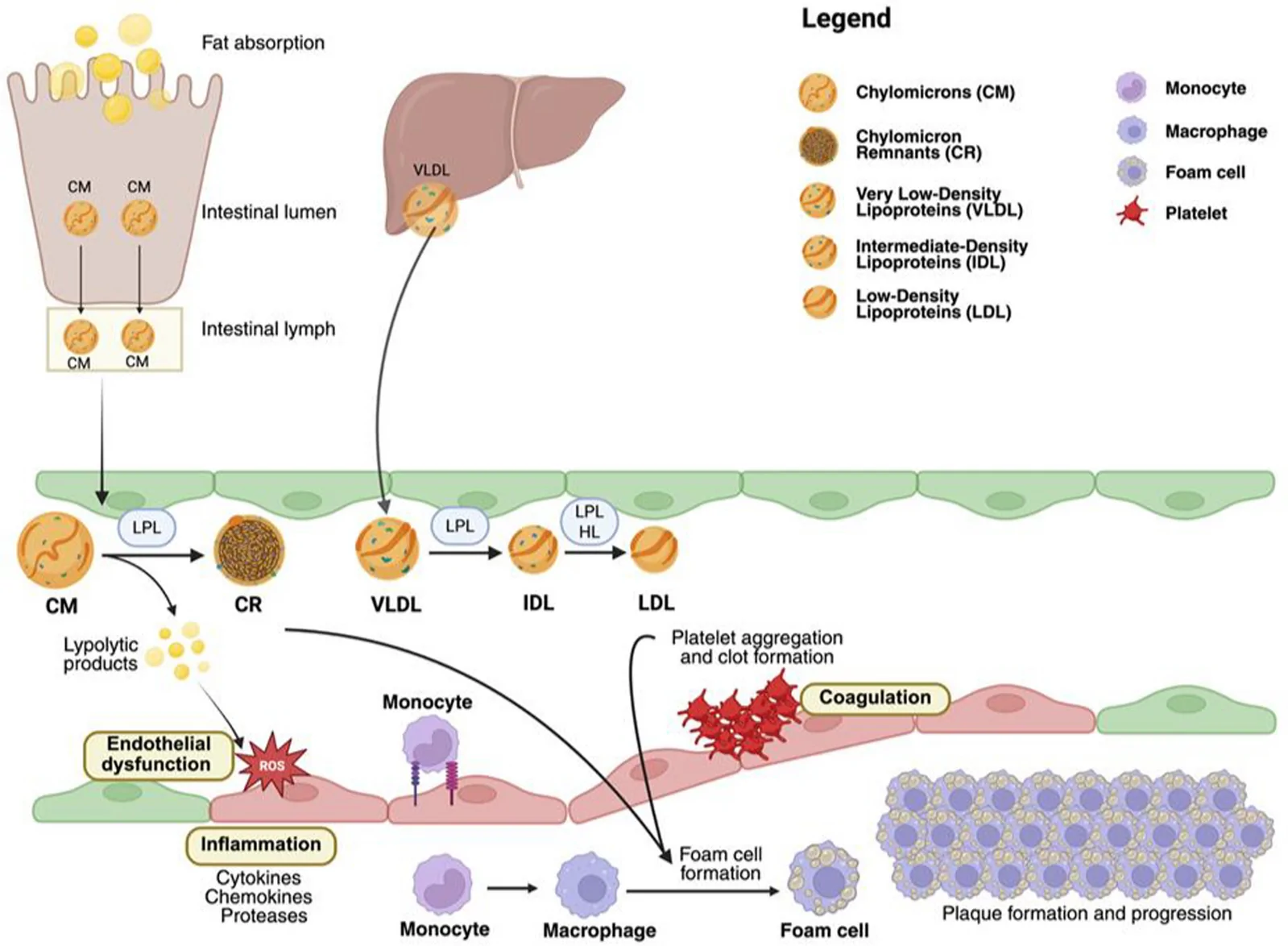
The multifaceted pathophysiology through which TRLs promote atherogenesis. Following intestinal fat absorption, TRLs, mainly CM, are secreted into the intestinal lymph and enter the circulation, while VLDL are produced by the liver. In the capillary bed, CM and VLDL undergo lipolysis mediated by LPL, leading to the release of lipolytic products and the formation of CR and IDL, which are further converted into LDL. TRL-derived lipolytic products promote ROS generation, resulting in endothelial dysfunction and activation of inflammatory pathways characterized by cytokine, chemokine, and protease release. Endothelial dysfunction facilitates monocyte recruitment and transmigration into the arterial wall, where monocytes differentiate into macrophages and internalize TRL-derived particles, giving rise to foam cells and contributing to plaque formation and progression. In parallel, TRL lipolysis products enhance platelet activation and aggregation, promoting coagulation and clot formation and thereby increasing atherothrombotic risk. *CM*, chylomicrons; *CR*, chylomicron remnants; *IDL*, intermediate-density lipoproteins; *LDL*, low-density lipoproteins; *LPL*, lipoprotein lipase; *ROS*, reactive oxygen species; *TRLs*, triglyceride-rich lipoproteins; *VLDL*, very-low-density lipoproteins

Figure 1 illustrates the multifaceted pathophysiology through which TRLs promote atherogenesis, from endothelial dysfunction to plaque instability.

Collectively, these mechanisms provide a strong biological basis for the atherogenic potential of TRLs. Building on this mechanistic understanding, extensive epidemiological and genetic evidence has further substantiated their causal involvement in atherosclerotic cardiovascular disease. Observational cohorts demonstrate that higher TG and remnant cholesterol predict cardiovascular events independently of LDL-C, while Mendelian randomization analyses confirm a causal relationship between lifelong TRL elevation and ASCVD. Key findings from major observational and genetic investigations are summarized in Tables 1 [34–54] and 2 [22, 55–66]. Collectively, both observational and genetic evidence converge to support a causal link between TRL/remnant cholesterol and atherosclerotic cardiovascular disease. This consistent signal across methodologies provides a strong rationale for therapeutic strategies aimed at reducing TRL burden through enhanced lipolysis, remnant clearance, or inhibition of key modulators such as APOC3 and ANGPTL3/4.

**Table 1:** Observational evidence linking triglycerides and remnant cholesterol to atherosclerotic cardiovascular disease risk

References	Population	Design	Key finding
Miller et al*.*, 2008 [34]	Post-ACS patients (n = 4,162)	RCT analysis	Lower on-treatment TG levels were associated with a lower risk of recurrent CHD events after ACS (HR 0.73)
Freiberg et al*.*, 2008 [35]	General population (n = 13,956)	Prospective cohort	Very high nonfasting TG were associated with an increased risk of ischemic stroke (men: HR 2.5; women: HR 3.8)
Varbo et al*.*, 2015 [36]	General population (n ~ 90,000)	Prospective cohorts	Extremely high RC (> 58 mg/dl) was associated with increased IHD (HR 2.4) and all-cause mortality risk (HR 1.6)
Joshi et al*.*, 2016 [37]	General population (n = 4932)	Pooled cohorts	Higher RLP-C was independently associated with incident CHD (HR 1.23 per 1-SD increase)
Castañer et al*.*, 2020 [38]	High-risk primary prevention (n = 6901)	Prospective cohort	Higher RC and atherogenic dyslipidemia were independently associated with incident CVD (HR 1.21 per 10 mg/dL and HR 1.44 respectively) and atherogenic dyslipidemia was also associated with increased risk
Wadström et al*.*, 2022 [39]	General population (n = 120,911)	Prospective cohorts	Elevated RC was associated with increased risk of PAD, MI, and ischemic stroke (PAD: HR 4.9; MI: HR 2.6; ischemic stroke: HR 2.1)
Hvid et al*.*, 2025 [40]	General population (n = 100,374)	Modelling study	Modeled RC reduction was associated with a projected reduction in 10-year ASCVD risk (absolute 10-year risk reduction 13–17% in women and 15–20% in men)
Drexel et al*.*, 2025 [41]	CAD patients (n = 1474)	Prospective cohort	Higher RC was associated with future all-cause mortality (HR 1.12), mortality for CVD (HR 1.2), and MACE (HR 1.1) in patients with established CAD
Jia et al*.*, 2024 [42]	General population (n = 327,899)	Prospective cohort	Higher TRLs-C was associated with increased MI risk across all ASCVD risk categories (HR 2.10 in the low-risk group, 1.52 in the intermediate-risk group, and 1.22 in the high-risk group)
Hokanson et al*.*, 1996 [43]	General population (n = 57,277)	Meta-analysis	Plasma TG were independently associated with CVD risk even after adjustment for HDL-C (men: RR 1.14; women: RR 1.37 per 1 mmol/L increase)
Quispe et al*.*, 2021 [44]	General population (n = 17,532)	Prospective cohorts	RC predicted CVD beyond LDL-C and apoB in primary prevention (HR 1.65)
Imke et al*.*, 2005 [45]	Middle-aged men (n = 1156)	Prospective cohort	RLP-C were independently associated with incident CHD in the Honolulu Heart Study
Kim et al*.*, 2015 [46]	General population (n = 86,476)	Prospective cohort	Higher TG levels were associated with increased cardiovascular event risk in Koreans (HR 2.40, highest vs lowest quartile)
Schwartz et al*.*, 2015 [47]	Post-ACS patients (n = 4162)	RCT analysis	Higher fasting TG predicted recurrent ischemic events after ACS despite statin treatment (HR 1.61 in the highest/lowest quintile (> 175/ ≤ 80 mg/dl)
Huang et al*.*, 2020 [48]	General population (n = 18,781)	Prospective cohort	Higher TG levels were associated with increased all-cause mortality, without evidence of a dose-dependent relationship
Balling et al*.*, 2020 [49]	General population (n = 109,751)	Observational + genetic	Higher VLDL-C was strongly associated with MI risk and accounted for about half of the apoB-related MI risk (HR 1.77 per 1 mmol/L higher VLDL-C)
Gao et al*.*, 2022 [50]	MINOCA patients (n = 1179)	Prospective cohort	High RC levels were significantly associated with an increased risk of MACEs after adjustment for multiple clinically relevant variables (per 1 SD increase, HR 0.61)
Huh et al*.*, 2022 [51]	T2D patients (n = 1.9 million)	Longitudinal cohort	Higher RC was associated with increased risk of MI and ischemic stroke in T2D (MI: HR 1.281; ischemic stroke: HR 1.22, highest vs lowest quartile)
Lee et al*.*, 2023 [52]	General population (n = 3.7 million)	Prospective cohort	Higher RC was independently associated with incident CVD among Koreans (HR 1.266)
Riis et al*.*, 2025 [53]	Older adults (70–100 yrs; n = 90,875)	Prospective cohort	Higher RC was associated with higher ASCVD incidence in healthy older adults (HR 1.31 per 1.0 mmol/L higher RC)
Aaseth et al*.*, 2025 [54]	Young adults (n = 5939)	Prospective cohort	Higher RC was associated with increased CVD events in young adults (aHR 1.3 per 0.5 mmol/L increase)

*ACS* acute coronary syndrome, *ASCVD* atherosclerotic cardiovascular disease, *CAD* coronary artery disease, *CHD* coronary heart disease, *CVD* cardiovascular disease, *HDL-C* high-density lipoprotein cholesterol, *IHD* ischemic heart disease, *LDL-C* low-density lipoprotein cholesterol, *MACE* major adverse cardiovascular events, *MI* myocardial infarction, *MINOCA* myocardial infarction with nonobstructive coronary arteries, *PAD* peripheral artery disease, *RC* remnant cholesterol, *RLP-C* remnant lipoprotein cholesterol, *RCT* randomized controlled trial, *TG* triglycerides, *TRL-C* triglyceride-rich lipoprotein cholesterol, *T2D* type-2 diabetes, *VLDL-C* very-low-density lipoprotein cholesterol

## Current therapeutic scenario

### Current recommendation (EAS/ESC guidelines)

European EAS/ESC guidance [67] defines LDL-C as the primary therapeutic target and addresses TG-related residual risk through secondary goals based on non-HDL-C or apoB when TG are elevated, with non-fasting sampling accepted for routine assessment; non-fasting TG are on average only ~ 0.3 mmol/L (27 mg/dL) higher and have comparable prognostic value. Indeed, in patients with elevated TG, diabetes, obesity, or very low LDL-C, the ESC/EAS guideline recommends using non–HDL-C or apoB for comprehensive risk assessment. Once LDL-C targets are achieved, secondary treatment goals may be applied: non–HDL-C < 2.2, < 2.6, and < 3.4 mmol/L for very-high-, high-, and moderate-risk patients, respectively, and apoB < 65, < 80, and < 100 mg/dL. Each non–HDL-C goal is set 0.8 mmol/L (30 mg/dL) above the corresponding LDL-C target.

Therapeutic management prioritizes lifestyle modification and correction of secondary causes. Fibrate therapy may be considered (Class IIb) as an adjunct to statins in high- or very-high-risk patients with persistent triglyceride levels ≥ 2.3 mmol/L (200 mg/dL), with the strongest evidence of benefit observed in those with the high-TG/low-HDL-C phenotype. However, the 2025 ESC/EAS Focused Update [9] emphasizes that consistent cardiovascular benefit has not been demonstrated in the overall population, and fibrate therapy should therefore be reserved for carefully selected patients with persistent hypertriglyceridaemia or features of atherogenic dyslipidemia despite optimal LDL-C lowering Table 2.

**Table 2:** Genetic evidence for the role of triglycerides and remnant cholesterol in atherosclerotic cardiovascular disease

References	Population	Method	Key finding
Varbo et al*.*, 2013 [22]	General population (n = 73,513)	MR	Genetically elevated RC (+ 1 mmol/L) was associated with higher IHD risk (OR 2.8); no causal association for HDL-C
Do et al*.*, 2013 [55]	Case–control cohorts (n ~ 87,000)	Polygenic analysis	Genetically determined TG (+ 1 SD) was associated with higher CAD risk (β = 0.21; P = 1 × 10⁻⁹), independent of LDL-C and HDL-C
Björnson et al*.*, 2023 [56]	UK Biobank (n ~ 487,000)	Multivariable MR	Genetically elevated TRL/RC (+ 1 mmol/L) was associated with higher CHD risk (OR 2.59), exceeding LDL-C (OR 1.37)
Navarese et al*.*, 2023 [57]	Consortia data (n ~ 958,000)	Two-sample MR	Genetically elevated RC (+ 1 SD) was associated with higher CAD risk (OR 1.51), MI (OR 1.57) and stroke (OR 1.23), independent of LDL-C
Zhao et al*.*, 2024 [58]	UK Biobank (n ~ 380,000–410,000)	MR	Genetically elevated RC (+ 0.29 mmol/L) was associated with higher cardiometabolic multimorbidity risk (OR 1.26); genetically elevated TG (+ 1 mmol/L) with OR 1.24
Khera et al*.*, 2017 [59]	Early-onset CAD cohorts (n ~ 47,000)	Gene-based study	Genetic variation in *LPL* was associated with higher TG (+ 19.6 mg/dL) and higher early-onset CAD risk (OR 1.84)
Crosby et al*.*, 2014 [60]	Multi-cohort population (n ~ 111,000)	Gene-based study	Genetic variation in *APOC3* was associated with lower TG (− 39%) and lower CHD risk (OR 0.60; 95% CI 0.47–0.75)
Nordestgaard et al*.*, 1997 [61]	General population (n ~ 9,000)	Gene-based study	*LPL* heterozygous deficiency was associated with higher TG (+ 0.8 mmol/L) and higher IHD risk (OR 4.9)
Jørgensen et al*.*, 2013 [62]	General population (n ~ 60,000)	MR	Genetic doubling of RC was associated with higher MI risk (OR 2.23); genetic doubling of TG with OR 1.94
Ibi et al*.*, 2022 [63]	UK Biobank + replication (n ~ 310,000)	Gene-based study	*APOA5*-associated genetically lower TG was associated with lower CAD risk (OR 0.95); combined APOA5 + LPL exposure OR 0.78
Zhou et al*.*, 2013 [64]	Case–control + meta-analysis (n ~ 12,000)	Gene-based study	*APOA5* − 1131 T > C was associated with higher CHD risk (OR 1.44)
Dewey et al*.*, 2016 [65]	DiscovEHR cohort (n ~ 43,000)	Exome sequencing	*ANGPTL4* LoF carriers had lower TG (− 13%) and lower CAD risk (OR 0.81) compared with noncarriers
Dewey et al*.*, 2017 [66]	DiscovEHR + replication (n ~ 58,000)	Exome sequencing	*ANGPTL3* LoF carriers had lower TG (− 27%) and lower CAD risk (OR 0.59) compared with noncarriers

*APOA5* apolipoprotein A5, *APOC3* apolipoprotein C3, *ANGPTL3* angiopoietin-like protein 3, *ANGPTL4* angiopoietin-like protein 4, *β* beta coefficient, *CAD* coronary artery disease, *CHD* coronary heart disease, *CI* confidence interval, *HDL-C* high-density lipoprotein cholesterol, *IHD* ischemic heart disease, *LDL-C* low-density lipoprotein cholesterol, *LPL* lipoprotein lipase, *LoF* loss-of-function, *MI* myocardial infarction, *MR* Mendelian randomization, *OR* odds ratio, *RC* remnant cholesterol, *SD* standard deviation, *TG* triglycerides, *TRL* triglyceride-rich lipoproteins

In the same update, particular attention is given to triglyceride-related residual risk. Icosapent ethyl 2 g twice daily in combination with a statin (Class IIa, Level B) is recommended for high- or very-high-risk patients with fasting triglycerides 135–499 mg/dL (1.52–5.63 mmol/L) to reduce cardiovascular events. For severe hypertriglyceridaemia due to familial chylomicronaemia, volanesorsen 300 mg weekly may be considered when triglycerides exceed 750 mg/dL (> 8.5 mmol/L) to mitigate pancreatitis risk, alongside strict dietary measures. Overall, the joint guideline recognizes TG/TRL-mediated risk as a modifiable component of residual cardiovascular risk, addressed pragmatically through non–HDL-C/apoB targets and selective TG-lowering therapies integrated with intensive LDL-C reduction, with non-fasting testing as the default approach.

The subsequent section systematically examines triglyceride-lowering therapies, focusing on pharmacological mechanisms, clinical trial evidence, and their role in contemporary cardiovascular prevention. Key pharmacological strategies targeting triglyceride-rich lipoproteins are compared in Table 3.

**Table 3:** Key pharmacological strategies targeting triglyceride-rich lipoproteins

Therapy	Mechanism of action	TG reduction (%)	Effect on apoB / non-HDL-C	Cardiovascular outcome data
Statins	↓ hepatic cholesterol synthesis (HMG-CoA reductase inhibition), ↑ LDLR, ↓ VLDL production	~ 10–30%	↓ apoB, ↓ non-HDL-C	Strong reduction in MACE (robust evidence)
Fibrates	PPAR-α activation → ↑ LPL activity, ↓ apoC-III, ↑ FA oxidation	~ 30–50%	↓ non-HDL-C, modest ↓ apoB	Neutral overall; benefit in high TG/low HDL subgroups
Omega-3 (EPA + DHA)	↓ VLDL synthesis, ↑ β-oxidation	~ 20–30%	Variable; possible ↑ LDL-C (DHA effect)	No consistent MACE reduction
Icosapent ethyl (EPA)	Anti-inflammatory, membrane stabilization, ↓ TG, ↓ oxidation	~ 20–30%	Minimal effect on apoB; ↓ non-HDL-C	↓ MACE (REDUCE-IT: − 25%)
MTP inhibitors (e.g., lomitapide)	Inhibits microsomal triglyceride transfer protein → ↓ VLDL assembly/secretion	~ 30–50%	↓ apoB (~ 39%) ↓ non-HDL-C (~ 40%)	No outcome data; limited by safety
FGF21 analogues (e.g., pegozafermin)	Improves hepatic lipid metabolism, ↑ insulin sensitivity, ↓ VLDL production	~ 30–60%	↓ non-HDL-C, ↓ apoB (moderate)	No outcome data
Evinacumab	Inhibits ANGPTL3 → ↑ LPL activity (partly LPL-independent)	~ 50–80%	Moderate to marked ↓ apoB and ↓ non-HDL-C	No outcome data yet
Volanesorsen (ASO)	↓ apoC-III → ↑ TRL clearance, ↓ hepatic VLDL	~ 77%	↓ apoB (~ 20%) ↓ non-HDL-C (~ 46%)	No outcome data yet
Olezarsen (ASO)		~ 60%	↓ apoB (~ 15%) ↓ non-HDL-C (~ 22%)	
Plozasiran (siRNA)		~ 60—80%	↓ apoB (~ 10—20%) ↓ non-HDL-C (~ 20%)	

*ANGPTL3* angiopoietin-like protein 3, *apoB* apolipoprotein B, *ASO* antisense oligonucleotide, *DHA* docosahexaenoic acid, *EPA* eicosapentaenoic acid, *FGF21* fibroblast growth factor 21, *HDL-C* high-density lipoprotein cholesterol, *LDL-C* low-density lipoprotein cholesterol, *LPL* lipoprotein lipase, *MACE* major adverse cardiovascular events, *MTP* microsomal triglyceride transfer protein, *non–HDL-C* non–high-density lipoprotein cholesterol, *PPAR-α* peroxisome proliferator-activated receptor alpha, *siRNA* small interfering RNA, *TG* triglycerides, *TRLs* triglyceride-rich lipoproteins, *VLDL* very-low-density lipoproteins

### Statins

While statins are primarily prescribed for their potent LDL-C-lowering effects, they also exert a modest yet clinically meaningful impact on TG levels and TRLs, making them foundational therapy in patients with high TG and elevated cardiovascular risk [68]. Several lines of evidence have consistently shown that statins reduce plasma TG concentrations, particularly in individuals with elevated baseline levels and comorbid metabolic conditions such as type 2 diabetes mellitus and obesity. The VOYAGER meta-analysis, which synthesized data from 32 randomized controlled trials involving over 37,000 patients, demonstrated that statin therapy leads to dose-dependent reductions in TG levels ranging from 10 to 30%, with greater reductions observed in those with baseline TG ≥ 200 mg/dL [69]. High-intensity statins, such as atorvastatin and rosuvastatin, were shown to be the most effective agents in this context. In the GREACE study [70], statin therapy with atorvastatin (10–80 mg/day) reduced LDL-C by 41% and TG by 28% in patients with metabolic syndrome, leading to improved cardiovascular outcomes. The JUPITER trial, which enrolled primary prevention patients with normal LDL-C but elevated hs-CRP, showed that rosuvastatin not only reduced LDL-C by 50% but also lowered TG by 17%, suggesting a dual benefit in patients with residual dyslipidemia [71]. Likewise, in the PROVE-IT TIMI 22 trial, intensive lipid-lowering with atorvastatin 80 mg reduced LDL-C by 51% and triglycerides by 20%, compared with 22% and 11% reductions with pravastatin (40 mg/day) and was associated with greater protection against death or major cardiovascular events [72].

From a mechanistic standpoint, statins decrease hepatic VLDL synthesis, upregulate LDL receptors, and enhance clearance of apoB-containing lipoproteins, including remnant particles [68]. These effects extend to non-HDL-C and apoB, which are key therapeutic targets in individuals with mixed dyslipidemia or HTG and reflect the burden of atherogenic TRLs. Moreover, statins possess pleiotropic properties, such as improving endothelial function, reducing vascular inflammation, and stabilizing atherosclerotic plaques, which further support their role in mitigating residual risk [73].

Current international guidelines, including the ESC/EAS [67] and ACC/AHA 2018 recommendations [74], endorse statins as the first-line pharmacologic intervention in patients with moderate hypertriglyceridemia (150–499 mg/dL), especially in high-risk or very-high-risk patients. The recent 2025 ESC/EAS Focused Update confirms this guidance, emphasizing the persistent role of statins as the cornerstone therapy for cardiovascular risk reduction in the presence of elevated TG, not only by targeting LDL-C but also by modulating TRLs and reducing the atherogenic potential of the lipid profile [9].

### Fibrates

Fibrates are a class of lipid-modifying agents first introduced in the late 1960s whose primary mechanism of action involves the activation of peroxisome proliferator-activated receptor alpha (PPAR-α), a nuclear receptor that regulates genes involved in lipid metabolism, including those encoding lipoprotein lipase, apolipoproteins A-I and A-II, and enzymes involved in fatty acid oxidation [75, 76]. This leads to enhanced catabolism of TRLs, increased HDL cholesterol synthesis, and decreased hepatic production of VLDL and apolipoprotein C-III, a natural inhibitor of lipoprotein lipase.

Among currently available pharmacological agents, fibrates remain one of the most effective therapies for achieving substantial reductions in fasting triglyceride levels. In fact, clinical studies have consistently demonstrated that fibrates reduce fasting TG levels by 30–50%, depending on baseline values and the specific agent used [77]. Fibrates are generally well tolerated, although hepatic, muscular, and renal adverse effects require clinical attention [78]. Transient elevations in liver transaminases and gastrointestinal symptoms are the most common adverse events, warranting periodic monitoring and treatment discontinuation in case of persistent abnormalities. Muscle-related side effects, ranging from myalgia to rare cases of rhabdomyolysis, may occur, particularly in the presence of concomitant statin therapy, renal impairment, hypothyroidism, or advanced age. This risk is markedly increased with gemfibrozil due to pharmacokinetic interactions, whereas fenofibrate is considered safer when combined with statins [78]. Fibrates may also induce reversible increases in serum creatinine, especially in patients with pre-existing renal impairment, likely reflecting functional changes in renal hemodynamics rather than structural injury [12, 79]. Consistently, creatinine elevations observed in the FIELD [77] and ACCORD-Lipid [80] trials resolved after treatment discontinuation and were not associated with markers of tubular damage. Moreover, fibrate therapy has been associated with reduced progression of albuminuria, supporting its use in patients with hypertriglyceridemia and early-stage chronic kidney disease [12], although the impact on long-term renal outcomes remains uncertain.

Beyond their effects on lipid metabolism and overall tolerability, the clinical relevance of fibrates has been extensively evaluated in terms of cardiovascular risk reduction, with heterogeneous results across clinical trials. Early trials, including the Helsinki Heart Study and the Veterans Affairs HDL Intervention Trial, showed significant reductions in coronary heart disease events with gemfibrozil [75, 76]. In contrast, later studies conducted largely in patients receiving statin therapy, such as FIELD [77] and ACCORD-Lipid [80], did not demonstrate a reduction in major cardiovascular events with fenofibrate in the overall study populations. Similar findings were reported in the PROMINENT trial, in which pemafibrate substantially lowered triglyceride levels without reducing major adverse cardiovascular events in patients with type 2 diabetes [81]. A subsequent meta-analysis suggested that any cardiovascular benefit associated with fibrates was largely explained by reductions in LDL cholesterol rather than triglycerides, helping to account for the difference between early and more contemporary trials [82]. These observations have led to a more selective view of fibrate therapy. Patients with atherogenic dyslipidaemia, particularly those with insulin resistance or type 2 diabetes, appear most likely to derive benefit. Patients with chronic kidney disease represent a high-risk population in whom hypertriglyceridemia is common, and fibrates may confer cardiovascular protection beyond their effects on lipids, as suggested by observational studies including a nested case–control analysis of pemafibrate use [83]. Overall, fibrates are highly effective triglyceride-lowering agents, but their impact on cardiovascular outcomes remains modest and confined to selected patient populations. Future studies should aim to refine patient selection and explore combination strategies targeting multiple lipid pathways.

Furthermore, emerging data also suggest that the effects of fibrates may extend beyond cardiovascular events, with reported benefits on diabetic retinopathy and metabolic liver disease, including hepatic steatosis [84]. Dedicated trials, including the Lowering Events in Non-Proliferative Retinopathy in Scotland (LENS) study, have demonstrated significant reductions in retinopathy progression with fenofibrate, with benefits observed even when glycemic control and traditional lipid measures are accounted for, raising the possibility of direct retinal effects independent of triglyceride lowering [84].

### *Omega-3 mixture (EPA* + *DHA)*

Omega-3 fatty acids are long-chain polyunsaturated fatty acids primarily obtained from marine sources, with two major bioactive forms: eicosapentaenoic acid (EPA) and docosahexaenoic acid (DHA). They exert their metabolic effects by reducing hepatic production of very-low-density lipoproteins (VLDL), enhancing β-oxidation, and modulating inflammatory signaling [85]. While some prescription products combine EPA and DHA, others contain purified EPA only, a distinction that has proven clinically relevant in outcome trials [86]. Over the last decade, large randomized controlled trials evaluating these different formulations have provided conflicting results on their effectiveness in reducing cardiovascular events, underscoring the importance of molecular composition and pharmacodynamics. Combined EPA + DHA formulations, such as omega-3 carboxylic acids and ethyl esters, effectively lower TG levels by reducing hepatic VLDL synthesis and enhancing fatty acid oxidation [87]. However, their impact on cardiovascular outcomes remains inconclusive. The STRENGTH trial (2020), which assessed omega-3 carboxylic acids (2.2 g EPA + 0.8 g DHA daily) in over 13,000 statin-treated patients at high cardiovascular risk with elevated TG, failed to demonstrate a significant reduction in MACE (HR 0.99; 95% CI 0.90–1.09; P = 0.84), leading to early termination for futility [88]. Potential explanations include the counterbalancing effects of DHA on LDL-C levels, suboptimal pharmacokinetics, or an unexpectedly active corn oil placebo. This neutral result is consistent with findings from several other large contemporary trials. In the ORIGIN trial, which enrolled more than 12,000 patients with impaired fasting glucose and at high risk for cardiovascular events, the administration of 1 g/day of combined EPA + DHA (465 mg of EPA and 375 mg of DHA) had no impact on cardiovascular mortality [89]. Similarly, both the ASCEND trial [90], conducted in 15,480 diabetic patients without known cardiovascular disease, and the VITAL study [91], which included > 25,000 healthy individuals, failed to show significant reductions in major cardiovascular events with EPA + DHA supplementation. Although results from the VITAL trial noted a modest reduction in myocardial infarction, the overall cardiovascular risk was unaffected [91]. These results are consistent with the pooled evidence from the meta-analysis by Khan et al. (38 randomized trials, n = 149,051), in which combination EPA + DHA therapy produced only modest but statistically significant reductions in cardiovascular mortality (RR 0.94; 95% CI 0.89–0.99; p = 0.02) and non-fatal myocardial infarction (RR 0.92; 95% CI 0.85–1.00; p = 0.04), substantially smaller than those observed with EPA monotherapy, underscoring the limited cardiovascular efficacy of mixed formulations [92]. A recent multicenter, double-blind randomized trial evaluated the effect of daily fish-oil supplementation (4 g of n − 3 polyunsaturated fatty acids [1.6 g of EPA and 0.8 g of DHA]) in patients on maintenance hemodialysis [93]. Over a median follow-up of 3.5 years, fish-oil significantly reduced the risk of major cardiovascular events, including myocardial infarction, stroke, and cardiovascular death, compared with placebo. The intervention was well-tolerated, though no significant difference in all-cause mortality was observed. These findings suggest that omega-3 supplementation may provide cardiovascular protection in a high-risk population with chronic kidney disease. From a mechanistic standpoint, omega-3 fatty acids are thought to exert anti-atherosclerotic effects not solely via TG lowering, but also by modulating vascular inflammation and endothelial function [94–96]. EPA and DHA are incorporated into phospholipid membranes of endothelial cells, where they influence membrane fluidity and receptor signaling [97]. These structural changes promote nitric oxide (NO) bioavailability and reduce endothelial expression of adhesion molecules such as VCAM-1 and ICAM-1, limiting monocyte adhesion and vascular inflammation [98]. Furthermore, omega-3 fatty acids serve as precursors to specialized pro-resolving lipid mediators, including resolvins and protectins, that actively promote resolution of inflammation within the vascular wall [99]. These mediators reduce cytokine production (e.g., IL-6, TNF-α), enhance macrophage phagocytosis, and inhibit neutrophil infiltration [100].

Despite these biologically plausible mechanisms, the inconsistent cardiovascular benefit observed with mixed EPA + DHA formulations may be attributed to the heterogeneity in patient populations, differences in omega-3 pharmacokinetics, and potential antagonism between EPA and DHA. Notably, DHA has been shown in some studies to modestly increase LDL-C levels, potentially offsetting some of EPA's anti-inflammatory and lipid-modifying benefits. These complex interactions underscore the need for more targeted therapeutic approaches and careful selection of both formulation and patient phenotype when considering omega-3 supplementation for cardiovascular prevention.

### Icosapent-ethyl

Icosapent ethyl (IPE) is a highly purified ethyl ester of EPA, specifically formulated to provide high plasma EPA concentrations without the inclusion of DHA. This compositional specificity is clinically meaningful, as trials of pure EPA have consistently demonstrated cardiovascular benefits that were not observed with combined EPA + DHA formulations. The clinical benefit of EPA monotherapy was first demonstrated by the JELIS study [101], which enrolled 18,645 hypercholesterolemic patients receiving statin therapy. Daily administration of 1.8 g of EPA led to a 19% relative reduction in MACE (p = 0.011), despite baseline TG levels within the normal range (median 153 mg/dL). Subsequently, the phase III studies, MARINE [102] and ANCHOR [103], further established the metabolic efficacy and safety profile of IPE. In MARINE study, conducted in patients with very high TG (≥ 500 mg/dL), IPE 4 g/day lowered TG levels by 33% without increasing LDL-C or ApoB, and significantly reduced large VLDL (28%) and small LDL (26%) particle concentrations. In ANCHOR study, which enrolled statin-treated patients with persistently high TG (200–499 mg/dL), IPE 4 g/day reduced TG by 22%, ApoB by 9%, and hs-CRP by 36%, while significantly increasing plasma EPA by more than six-fold and lowering the arachidonic-acid/EPA ratio by 91%. These findings established that IPE favorably modulates atherogenic lipoprotein profiles and systemic inflammation without raising LDL-C, differentiating it mechanistically from DHA-containing omega-3 preparations. The cardiovascular efficacy of IPE was robustly demonstrated in the REDUCE-IT trial, which enrolled 8,179 statin-treated patients with either established cardiovascular disease or diabetes plus additional risk factors, and fasting TG levels between 135 and 499 mg/dL [104]. Participants were randomized to receive 4 g/day of IPE or a mineral oil placebo. Over a median follow-up of 4.9 years, IPE significantly reduced the risk of MACE by 25% (HR 0.75; 95% CI 0.68–0.83; p < 0.001), with consistent reductions across all major components, including cardiovascular death, myocardial infarction, stroke, and coronary revascularization. The absolute risk reduction was 4.8%, yielding a number needed to treat (NNT) of 21. Notably, the magnitude of benefit observed in REDUCE-IT exceeded that expected from triglyceride lowering alone [104]. In support of this interpretation, a formal mediation analysis of REDUCE-IT demonstrated that increases in plasma EPA, together with reductions in arachidonic acid, accounted for most of the observed cardiovascular benefit, whereas triglyceride lowering contributed to a substantially smaller proportion of the treatment effect. Specifically, EPA alone mediated approximately 57% of the risk reduction, and EPA, arachidonic acid, and triglycerides jointly explained nearly 80% of the total treatment effect on MACE [105]. Beyond lipid modification, biomarker analyses from REDUCE-IT [104] demonstrated that IPE significantly reduced systemic inflammation and oxidative stress, including a 39.9% decrease in hs-CRP, a 13% reduction in oxidized LDL, and an 18% decrease in lipoprotein-associated phospholipase A₂ compared with minimal changes in the placebo group. These effects were proportional to achieved plasma EPA concentrations rather than TG lowering, underscoring EPA’s pleiotropic effects. The mechanistic plausibility of these effects was further supported by imaging and clinical outcome studies. It is worth emphasizing that the use of mineral oil as placebo in REDUCE-IT has raised concerns, as it may have interfered with statin absorption and contributed to modest increases in LDL-C and inflammatory markers in the control group. However, these changes were relatively small and unlikely to account for the magnitude of the observed risk reduction. Consistently, post hoc analyses and findings from the JELIS trial [101], which did not use a mineral oil placebo, support a genuine cardiovascular benefit of EPA therapy. In the EVAPORATE trial [106], IPE 4 g/day added to statin therapy significantly slowed and partially reversed coronary atherosclerosis progression, reducing low-attenuation plaque volume by 17% compared with a 109% increase in the placebo group (p = 0.006), along with reductions in total and fibrofatty plaque components. Similarly, the RESPECT-EPA trial [107], conducted in statin-treated Japanese patients with stable coronary artery disease and a low baseline EPA-to–arachidonic acid ratio, showed a numerically lower incidence of major cardiovascular events with icosapent ethyl that did not reach statistical significance for the primary end point, although a significant reduction was observed in the secondary composite of coronary events over five years of follow-up.

These results have renewed debate on the heterogeneous efficacy of omega-3 formulations, given the contrasting findings of the STRENGTH trial [88]. In REDUCE-IT [104], the use of a purified EPA ethyl-ester formulation led to substantially higher plasma EPA concentrations (~ 144 µg/mL) than those achieved in STRENGTH (~ 90 µg/mL), a difference that appears critical for anti-inflammatory and membrane-stabilizing activity. Moreover, the absence of DHA in IPE avoids potential counteractive effects of DHA on LDL-cholesterol and membrane structure, which may have attenuated efficacy in the mixed formulation tested in STRENGTH. Finally, differences in molecular form ethyl ester versus carboxylic acid could further influence absorption, bioavailability, and membrane incorporation, collectively accounting for the distinct biological responses observed between the two studies.

Collectively, these data establish IPE as the only omega-3–derived therapy with consistent and reproducible cardiovascular benefit, supported by mechanistic evidence of anti-inflammatory, antioxidant, and plaque-stabilizing actions that extend beyond TG lowering.

## Novel emerging therapies

**Fig. 2:**
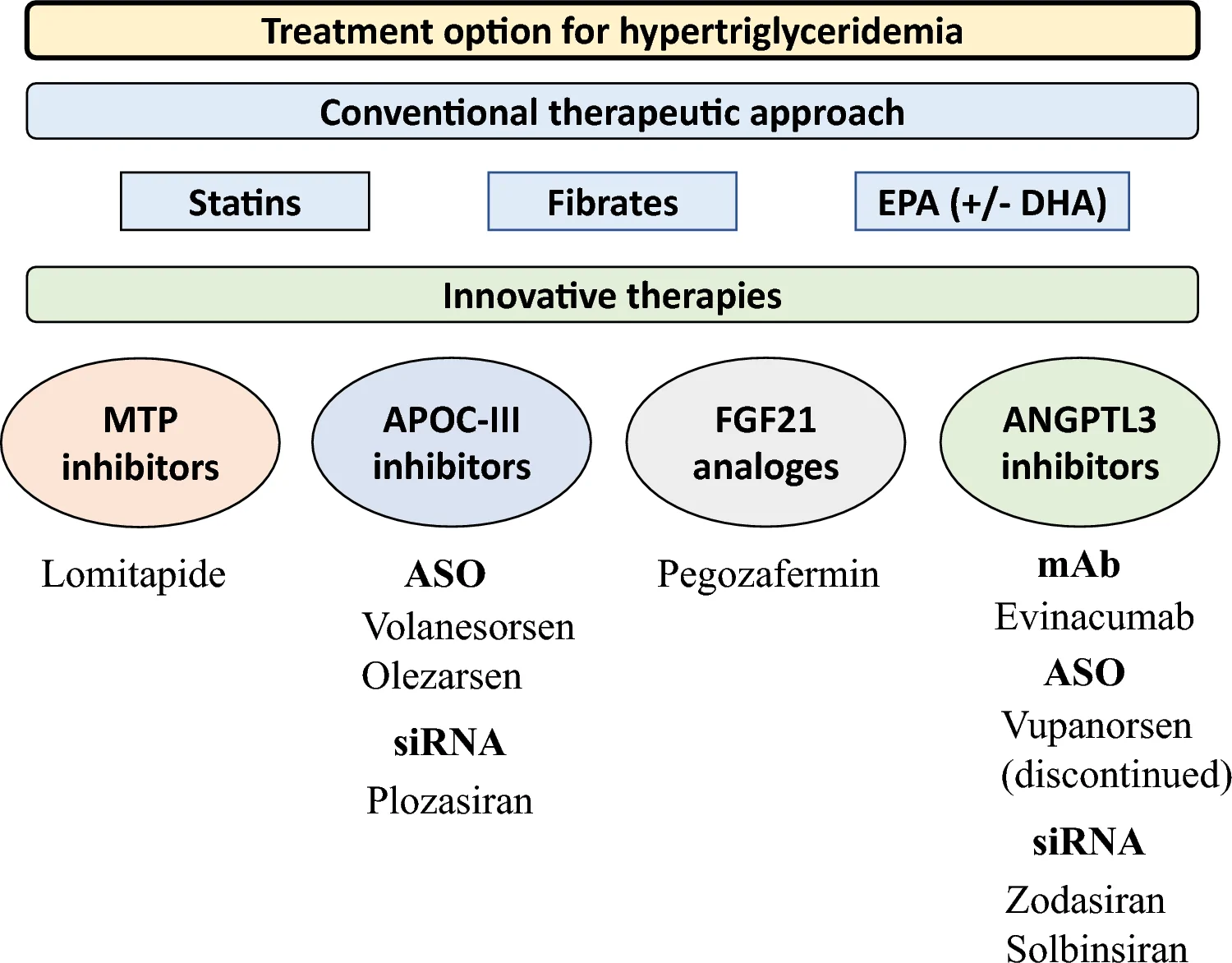
Current and emerging therapeutic options for hypertriglyceridemia. Established therapeutic approaches include statins, fibrates, and omega-3 fatty acids (EPA ± DHA). Emerging therapeutic strategies target key regulators of TG metabolism and include MTP inhibitors, APOC-III inhibitors (ASO and siRNA), FGF21 analogues, and ANGPTL3 inhibitors, comprising mAb-, ASO-, and siRNA-based agents. *ANGPTL3*, angiopoietin-like protein 3; *APOC-III*, apolipoprotein C-III; *ASO*, antisense oligonucleotide; *DHA*, docosahexaenoic acid; *EPA*, eicosapentaenoic acid; *FGF21*, fibroblast growth factor 21; *mAb*, monoclonal antibody; *MTP*, microsomal triglyceride transfer protein; *siRNA*, small interfering RNA; *TG*, triglycerides

The therapeutic landscape for severe HTG is undergoing a paradigm shift with the development of targeted agents acting on key regulators of TG metabolism (Fig. 2). Although current evidence for these emerging therapies derives primarily from studies in patients with chylomicronemia syndromes and pancreatitis prevention, their profound effects on TG metabolism and remnant lipoproteins suggest potential applications in mixed dyslipidemias and atherogenic residual risk. Among emerging lipid-lowering agents, evinacumab, a fully human monoclonal antibody against ANGPTL3, has shown compelling efficacy in reducing TG levels and addressing residual cardiovascular risk in select patient populations. In a phase 2 trial (NCT03452228) [108], intravenous evinacumab 15 mg/kg every 4 weeks resulted in highly variable, but sometimes marked TG reductions, up to 70–80%, in patients with multifactorial chylomicronemia without *LPL* loss-of-function variants, whereas modest effect was observed in those with complete LPL deficiency. A subsequent phase 2b study (NCT04863014) [109] in patients with severe HTG and a history of pancreatitis confirmed a substantial median TG decrease of 55% at week 4 and > 90% at week 16 with the 20 mg/kg dose, together with consistent improvements in non-HDL cholesterol and ApoC-III levels. Importantly, evinacumab acts through mechanisms at least partly independent of LPL activity and was well tolerated, with lower rates of adverse events than placebo. Beyond triglyceride lowering, evinacumab favorably influences non-HDL cholesterol, ApoB, and remnant lipoproteins, factors closely linked to atherogenesis. These broader lipid improvements suggest a potential role in cardiovascular risk reduction, particularly in patients with mixed dyslipidemia or persistently elevated TRLs despite statin therapy and this is supported by human genetic data linking ANGPTL3 deficiency to lower incidence of atherosclerotic cardiovascular disease [110].

Another established drug target for novel lipid-lowering strategies is represented by Apolipoprotein C-III (apoC-III), a key modulator of TRLs metabolism. Elevated apoC-III levels are associated with increased circulating TG, impaired remnant clearance, and a heightened burden of atherogenic lipoproteins. Indeed, beyond its inhibitory action on lipoprotein lipase (LPL), apoC-III also delays hepatic uptake of TRL remnants, contributing to both hypertriglyceridemia and atherosclerotic progression through LPL-independent pathways [111]. Mendelian randomization studies have provided strong genetic evidence that loss-of-function variants in APOC3 confer protection against coronary artery disease, independent of LDL cholesterol levels [60, 112], suggesting that apoC-III inhibition may represent a valid therapeutic strategy for ASCVD. Although apoC-III has long been recognized as a regulator of triglyceride metabolism and is modestly reduced by conventional therapies such as fibrates, the degree of suppression achieved with these agents is limited and has not translated into consistent cardiovascular benefit [113]. For this reason, novel therapeutic strategies have been developed to achieve more potent and selective inhibition of apoC-III synthesis. Volanesorsen, a second-generation antisense oligonucleotide (ASO) targeting hepatic APOC3 mRNA, has shown the capacity to lower TG by up to 77% in patients with Familial chylomicronemia syndrome (FCS) [114]. However, despite its metabolic efficacy, volanesorsen has not been evaluated in cardiovascular outcome trials, and its safety profile, particularly the high incidence of thrombocytopenia, has limited broader clinical adoption [115]. Furthermore, most available studies have been conducted in rare monogenic disorders, which may not reflect the mechanisms underlying residual cardiovascular risk in broader dyslipidemic populations. Olezarsen, a next-generation GalNAc-conjugated ASO targeting hepatic APOC3 mRNA, achieves potent, durable, and hepatocyte-selective inhibition of apoC-III synthesis. In the phase 3 BALANCE trial [116], conducted in patients with FCS, olezarsen 80 mg reduced apoC-III by 74% and TG by 43% at 6 months, reaching 59% at 12 months, while markedly lowering the incidence of acute pancreatitis compared with placebo (rate ratio 0.12; 95% CI 0.02–0.66). In the ESSENCE–TIMI 73b trial [117], which enrolled over 1300 statin-treated patients with moderate HTG, olezarsen produced placebo-adjusted reductions of approximately 60% in triglycerides and 70% in apoC-III, along with significant decreases in non–HDL cholesterol (− 22%) and apoB (− 15%) and increases in HDL-C (+ 46%), without raising LDL-C. These data highlight the consistency of effect across metabolic phenotypes, indicating that potent apoC-III inhibition benefits both monogenic and polygenic forms of hypertriglyceridemia through mechanisms extending beyond LPL activation and confirm olezarsen’s broad metabolic efficacy, consistent safety, and potential for reducing both pancreatitis and residual atherosclerotic risk. Plozasiran, a GalNAc3-conjugated small interfering RNA (siRNA) that silences hepatic APOC3, has demonstrated comparable efficacy with longer dosing intervals. In the phase 3 PALISADE trial [118], plozasiran reduced apoC-III by ~ 90% and TG by 78–80% at 10 months, achieving TG levels < 500 mg/dL in about half of patients and < 150 mg/dL in over 90% of those with partial LPL activity. In MUIR [119] and SHASTA-2 [120], plozasiran similarly reduced TG by 50–60% and improved remnant cholesterol, non–HDL-C, and apoB, while modest, dose-dependent LDL-C increases were observed at higher doses. In summary, apoC-III represents a biologically compelling target linking triglyceride-rich lipoproteins, remnant clearance, and atherosclerotic risk. While prior pharmacological approaches, including fibrates, have achieved only modest apoC-III suppression without consistent cardiovascular benefit, next-generation antisense and siRNA-based therapies enable profound and sustained inhibition of apoC-III. Whether this degree of target engagement will translate into reductions in cardiovascular events remains to be established in dedicated outcome trials.

Finally, other emerging approaches, including lomitapide, a microsomal triglyceride transfer protein inhibitor, and pegozafermin, an FGF21 analog with combined triglyceride and hepatic fat–lowering properties, may further expand therapeutic options for complex hypertriglyceridemia [121]. However, their role in atherosclerotic cardiovascular prevention awaits confirmation from adequately powered outcome studies.

## Conclusion

Triglycerides and their remnant lipoproteins are now recognized as causal contributors to residual cardiovascular risk, even when LDL-C is optimally controlled. Risk assessment should therefore look beyond LDL-C to include TG, non-HDL-C/apoB, and remnant cholesterol, alongside rigorous correction of secondary causes. Taken together, although data for triglyceride-lowering therapies remain heterogeneous and often controversial, icosapent ethyl is the only intervention to date that has demonstrated a robust reduction in major cardiovascular events, whereas emerging TRL-targeted agents (e.g., APOC3 and ANGPTL3 inhibitors) show impressive metabolic and pancreatitis benefits but still require adequately powered cardiovascular outcome trials to define their role in risk reduction.

## Data Availability

No new datasets were generated or analyzed during the current study.
